# Membership in the American Medical Association

**Published:** 1891-09

**Authors:** 


**Membership in the American Medical Association.**—This is obtainable, at any time, by a member of any State or local Medical Society which is entitled to send delegates to the Association. All that is necessary is for the applicant to write to the Treasurer of the Association, Dr. Richard J. Dunglison, Lock Box 1274, Philadelphia, Pa., sending him a certificate or statement that he is in good standing in his own Society, signed by the President and Secretary of said Society, with five dollars for annual dues. Attendance as a delegate at an annual meeting of the Association is not necessary in order to obtain membership. On receipt of the above amount the weekly Journal of the Association will be forwarded regularly.

